# Gene ssa-miR-301a-3p improves rainbow trout (*Oncorhynchus mykiss*) resistance to heat stress by targeting *hsp90b2*

**DOI:** 10.7717/peerj.13476

**Published:** 2022-07-05

**Authors:** Zhe Liu, Fang Ma, Yujun Kang, Xiaoxia Liu

**Affiliations:** 1 Gansu Agricultural University, Lanzhou, China; 2 Tianshui Normal University, Tianshui, China

**Keywords:** Expression regulation, ssa-miR-301a-3p, hsp90b2, HEK 293T cells, Rainbow trout

## Abstract

Rainbow trout (*Oncorhynchus mykiss*) is a cold-water fish that is commonly harmed by high temperatures. MicroRNAs (miRNAs) are being investigated intensively because they act as essential metabolic regulators and have a role in the heat stress response. Although there have been numerous studies on rainbow trout heat stress, research on miRNA implicated in rainbow trout heat stress is quite restricted. Rainbow trout were sampled at 18 and 24 °C, respectively, to examine the mechanism of miRNA under heat stress, and we identified a heat stress-induced miRNA, ssa-miR-301a-3p, for further investigation based on our bioinformatics analysis of rainbow trout small RNA sequencing data. Bioinformatics research suggested that *hsp90b2* is a probable target gene for ssa-miR-301a-3p. QRT-PCR was used to confirm the expression levels of ssa-miR-301a-3p and *hsp90b2*. Meanwhile, the dual-luciferase reporter assay was employed to validate the ssa-miR-301a-3p*-hsp90b2* targeted connection. The results indicated that at 24 °C, the relative expression of ssa-miR-301a-3p was considerably lower than at 18 °C. On the other hand, *hsp90b2* expression, followed the opposite pattern. The binding of ssa-miR-301a-3p to the 3′-UTR of *hsp90b2* resulted in a substantial decrease in luciferase activity. The findings showed that ssa-miR-301a-3p was implicated in heat stress, and our findings provide fresh insights into the processes of miRNA in response to heat stress in rainbow trout.

## Introduction

Clusters consisting of 21–23 nucleotides and single-stranded non-coding small RNAs are called miRNAs. They can specifically bind to the 3′-untranslation regions (3′-UTRs) of their target genes by a base pair-mediated fashion, and negatively regulate downstream target gene expression at the post-transcriptional level (Bartel, 2004; Baulcombe, 2004; Carrington & Ambros, 2003; Chen & Meister, 2005; Lee & Ambros, 2001; Matzke et al., 2004). In recent years, a number of studies have focused on the relationship between miRNA and temperature stresses, and many studies have demonstrated that the expression levels of miRNA are regulated by temperature stress (Yu et al., 2010; Zhang et al., 2014; Zhang et al., 2017). The expressions of miR-199a-3p and miR-34a-5p are most significantly different between heat-tolerant (TOL) mice and heat-intolerant (INT) mice. Other miRNAs including miR-19a, miR-19b, miR-27b, miR-30a-5p, miR-181a, miR-181b, miR-345-3 and miR-1246 were found in the serum of Holstein cows under heat stress (Zheng et al., 2014). Moreover, miR-23a is involved in heat stress, miR-23a can regulate the expression of *CDK5*, and the decrease of miR-23a expression increases the expression of *CDK5* protein to resist heat stress (Morey et al., 2015).

As a member of the miR-130 family, ssa-miR-301a-3p is highly conserved in rats, chickens and cattle and can promote the proliferation, migration and angiogenesis of vascular endothelial cells (Zhou et al., 2012). After water temperature increased, miR-301 expression in gonads of zebrafish was significantly down-regulated (Ikert & Craig, 2020). In the carp study, miR-301a in the liver was found to regulate the perception of cellular nutrients, oxygen, energy levels, and the activation of insulin receptors and insulin-like growth factor 1 receptors when affected by temperature (Sun et al., 2019). In fish, miR-301a-3p is also implicated in the regulation of oxidative stress, inflammation, and apoptosis, the expression level of miR-301a-3p is significantly up-regulated under hypoxia pressure (Jiang & Lv, 2021; Xia et al., 2020). Intriguingly, the expression of miR-301a-3p, as well as the expressions of other miR-130a family members including miR-130b-3p, miR-130a-3p, and miR-301b-3p, were significantly upregulated by heat stress (Zhang et al., 2020). Taken together, data above may suggest a potential relationship among miR-301a-3p and heat stress. Although hundreds of miRNAs have been identified since the discovery of the first member 20 years ago (Bushati & Cohen, 2007), studies on the function of miRNAs in animals under heat stress are limited, especially fish. Based on our bioinformatics analysis of small RNA sequencing data of rainbow trout (Huang et al., 2018), we identified one miRNA, ssa-miR-301a-3p, which was significantly down-regulated after heat stress. In this study, our aim was to verify that ssa-miR-301a-3p is involved in heat stress in rainbow trout. We predicted the target gene of ssa-miR-301a-3p by using bioinformatics analysis to screen RNA-seq data (Li et al., 2017).

Because global warming threatens animal health and animal husbandry economy, heat stress, as one of the important abiotic stresses, interferes with cellular homeostasis and induces physiological and biochemical changes in animals (St-Pierre, Cobanov & Schnitkey, 2003; Quinteiro-Filho et al., 2010). Exposure to high temperatures may alter gene expression, leading to the synthesis of stress-related proteins, such as heat shock proteins (HSPs), which are known as key regulators in the heat stress response (Iba, 2002) and will be up-regulated when the organism suffers high temperatures (Lindquist, 1986). HSPs are highly conservative proteins that are induced when the organism is affected by virus infection, temperature variation, heavy metals, starvation and osmotic shock, hypoxia, and oxidative stress in the environment (Haase & Fitze, 2016; Basu et al., 2002; Cara et al., 2005; Kregel, 2002). Heat shock protein 90 (hsp90) is involved in protein sorting, maturation, stabilization and activation (Jackson, 2013; Hong et al., 2013). In vertebrates, *hsp90* has six major isoforms (*hsp90a1a*, GenBank accession no. KC150880. *hsp90a1b*, GenBank accession no. KC150881. *hsp90a2a*, GenBank accession no. KC150878. *hsp90a2b*, GenBank accession no. KC150879. *hsp90b1*, GenBank accession no. KC150882 and *hsp90b2*, GenBank accession no. KC150883), and a previous study showed that mRNA level of *hsp90b2* in rainbow trout hepatocytes was significantly increased after heat shock (Jia, Cavileer & Nagler, 2016).

Rainbow trout is a major commercial and cold-water fish species and has a preferred water temperature. The appropriate growth temperature for rainbow trout is 12–18 °C, and the water temperature above 25 °C is perilous (Matthews & Berg, 1997). Our previous studies have found that the growth and development of rainbow trout will be severely affected when water temperature increases (Xia et al., 2017). High temperatures can lead to economic losses due to lower productivity and reproductivity, as well as increased health burden. Heat stress is one of the most characteristic environmental stressors. Individuals respond to heat stress by increasing body temperature, reducing feed intake and changing physiological state, which is called physiological adaptation response. However, other protective measures have been developed in animals to cope with the challenges of heat stress, mainly manifested as altered transcription and gene expression levels. Our previous analysis showed that 24 °C is a key high-temperature point that the organism changed from adaptive regulation to injury (Xia et al., 2018). Thus, we chose 18 and 24 °C as the control and heat treatment temperature, respectively. In this study, we are investigating miRNAs that are induced at optimal (18 °C) and high (24 °C) temperatures in the liver of rainbow trout (Huang et al., 2018). We used bioinformatics software to predict their target genes and found that miR-301a-3p regulate *hsp90b2*. Herein our core objective was to validate if *hsp90b2* is indeed the target genes of miR-301a-3p, and for this purpose, we used the Dual-Luciferase® Reporter Assay System. Subsequently, we combined the results of Gene Ontology (go) and Kyoto Encyclopedia of genes and genomes (KEGG) pathway enrichment (Huang et al., 2018) to analyze the main biological functions of the miR-301a-3p and *hsp90b2*, and to evaluate the related regulatory mechanisms. The objective of this study was to validate targets of ssa-miR-301a-3p and evaluate the possible role of ssa-miR-301a-3p in the liver of rainbow trout under heat stress by repressing *hsp90b2*. The results of this study will provide useful information of miRNA in response to heat stress in rainbow trout liver, and provide a reference for the studies of heat stress of this species in the future.

## Materials and Methods

### Ethical approval

This study was conducted in strict accordance with the guidelines for the Care and Use of Laboratory Animals in China and was approved by the Institutional Ethic Committee of Gansu Agricultural University.

#### *Oncorhynchus mykiss* preparation

Healthy rainbow trout (average weight: 400 ± 10.5 g) belonging to full-sibling inbred families were obtained from the trout cage aquafarm in Liujiaxia reservoir (Gansu, China) and temporarily sustained in a 3,000 L aerated water tank at 18 °C for 1 week. A total of 120 healthy fish were chosen for subsequent experiments. Those fish were randomly divided into six groups and followed by an adaptation for another 7 days at 18 °C. Three groups were maintained at 18 °C and the other three groups were treated with a thermal stress. The temperature system continued to increase with a heating rate of 1 °C/24 h from 18 to 24 °C. After anesthetizing the fish with a lethal dose of MS-222, the liver samples were harvested from female fish at 18 (control group, CL) and 24 °C (heat stress group, HL) respectively. The tissues were immediately flash frozen in liquid nitrogen, and stored at −80 °C until RNA extraction.

### Target genes prediction

Based on our miRNA-seq data, we found that the expression level of one miRNA, ssa-miR-301a-3p, was significantly downregulated in rainbow trout livers under heat stress. In order to understand the mechanism of ssa-miR-301a-3p, the potential target genes of ssa-miR-301a-3p were predicted by using TargetScan (www.targetscan.org), PicTar (https://pictar.mdc-berlin.de/), and miRanda (https://cbio.mskcc.org/miRNA2003/miranda.html) analysis of RNA-seq data, which was the result of our previous research group (Li et al., 2017).

### Quantitative real-time RT-PCR analysis

For the ssa-miR-301a-3p, the first-strand cDNA was synthesized from the total RNA using Mir-X miRNA First-Strand Synthesis Kit (TaKaRa, Dalian, China). Specific primer for the ssa-miR-301a-3p was designed and primer sequences were listed in Table 1. The qRT-PCR was performed using Mir-X miRNA qRT-PCR SYBR Kit (TaKaRa, Dalian, China) and LightCycler® 480 Instrument II (Roche, Basel, Switzerland). *U6* was used as an endogenous control. The cycling parameters were as follows: 95 °C for 10 s, 40 cycles at 95 °C for 5 s and 60 °C for 20 s; melting curve analyses at 95 °C for 60 s, 55 °C for 30 s, and 95 °C for 30 s.

**Table 1:** Primers used for the quantitative real-time PCR (qRT-PCR).

Gene name	Accession no.	Primer sequence (5′ to 3′)
*Hsp90b2*	KC150883	F: 5′-GTTTGGCGTGGGTTTCTACTCT-3′
		R: 5′-CCACCTCCTTGACCCTCTTCT-3′
*ACTB*	AB196465	F: 5′-TGGGGCAGTATGGCTTGTATG-3′
		R: 5′-CTCTGGCACCCTAATCACCTCT-3′
Ssa-miR-301a-3p	MIMAT0032593	F: 5′-AGTGCCATAGTATTGTCATAGC-3′
		R: mRQ 3′primer
*U6*	XM_021614042	F: 5′-GGAACGATACAGAGAAGATTAGC-3′
		R: 5′-TGGAACGCTTCACGAATTTGCG-3′

**Note:**

F, forward; R, reverse.

For *hsp90b2*, the PrimerScript™RT Reagent Kit with gDNA Eraser (TaKaRa, Dalian, China) was used to synthesize first-strand cDNA, and SYBR® Premix Ex Taq kit (TaKaRa, Dalian, China) and LightCycler® 480 Instrument II were used for qRT-PCR. The *ACTB* was taken as a reference gene. Specific primer for the *hsp90b2* was listed in Table 1. The cycling parameters were as follows: 95 °C for 10 s, 40 cycles at 95 °C for 5 s and 61 °C for 20 s; melting curve analyses at 95 °C for 60 s, 55 °C for 30 s, and 95 °C for 30 s.

### Construction of recombinant plasmid

**Figure 1:**
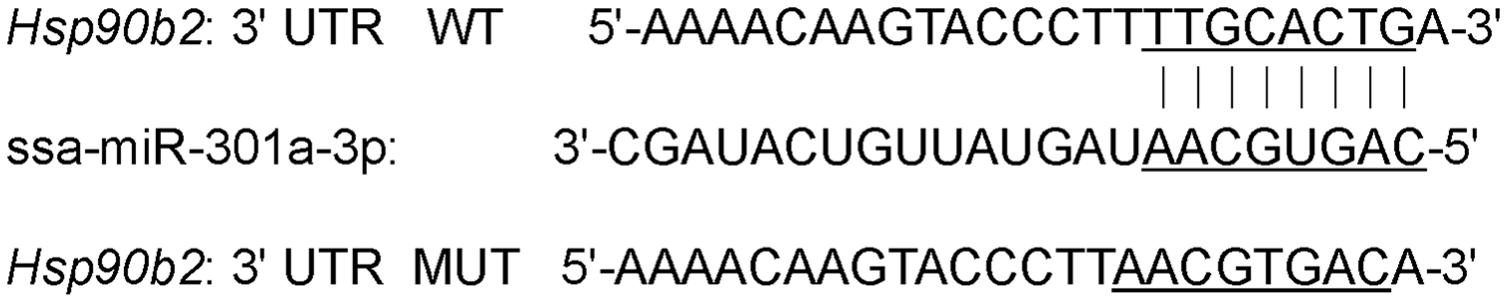
3′-UTR binding site of *hsp90b2* mRNA and ssa-miR-301a-3p.

3′-UTR segment of the *hsp90b2* with the ssa-miR-301a-3p binding site was cloned into pmirGLO dual-luciferase vector (Promega, Madison, WI, USA), which was digested with restriction enzymes *SacI* and *XhoI*, and produced a wild-type structure pmirGL0-hsp90b2-wt-3′-UTR (hsp90b2 WT) by using ClonExpress® Entry One Step Cloning Kit (GenePharma, Shanghai, China). 3′-UTR mutation sequence of *hsp90b2* pmirGLO-hsp90b2-mut-3′-UTR (hsp90b2 MUT) constructed by binding site (TTGCACTG) within the 3′-UTR of the *hsp90b2* was changed using Site-Directed Mutagenesis Kit (Beyotime, ShangHai, China) and took a place of (AACGTGAC) (Fig. 1). At the same time, reverse complementary sequences of ssa-miR-301a-3p were cloned to pmirGLO dual-luciferase vector to construct positive control (ssa-miR-301a-3p PC), which was transferred to HEK 293T cells and was processed to siRNA (small interference RNA) segments of 21–23 bp in length by Dicer, and then siRNA recognized ssa-miR-301a-3p mimic with homologous sequences through base complementary ligation and mediated ssa-miR-301a-3p mimic degradation. All recombinant plasmids were tested for enzyme digestion and sequencing.

### Cell culture and transfection

HEK 293T cells were purchased from cell Bank of Chinese Academy of Sciences. HEK 293T cells were cultured in Dulbecco’s Modified Eagle Medium (DMEM) containing 10% fetal bovine serum (FBS) at 37 °C with 5% CO_2_ saturated humidity incubator. Data were collected as previously described in Liu et al. (2021), ssa-miR-301a-3p mimic and mimic negative-control (NC) were synthesized from GenePharma (Shanghai, China). The sequences are as follows: ssa-miR-301a-3p mimic sense: 5′-CAGUGCAAUAGUAUUGUCAUAGC-3′, ssa-miR-301a-3p mimic antisense: 5′-UAUGACAAUACUAUUGCACUGUU-3′; mimic NC sense: 5′-UUCUCCGAACGUGUCACGUTT-3′, mimic NC antisense: 5′-ACGUGACACGUUCGGAGAATT-3′.

The transfection group consisted of mimic NC + hsp90b2 WT, mimic NC + hsp90b2 MUT, ssa-miR-301a-3p mimic + hsp90b2 WT, ssa-miR-301a-3p mimic + hsp90b2 MUT, mimic NC + ssa-miR-301a-3p PC, and ssa-miR-301a-3p mimic + ssa-miR-301a-3p PC.

### Dual-luciferase reporter assay

The HEK 293T cells were seeded at a density of 5.0 × 10^5^ cells per well in a 12-well culture plate, incubated for 24 h before transfection, and then transfected with 10 μl of ssa-miR-301a-3p mimic, 10 μl of mimic NC (GenePharma, Shanghai, China), 1.6 μg of hsp90b2 WT, 1.6 μg of hsp90b2 MUT and 1.6 μg ssa-miR-301a-3p PC plasmid simultaneously. Meanwhile, a total of 4 μl of Lipofectamine 2000 (Invitrogen, Waltham, MA, USA) was added to another Eppendorf tube containing 100 μl of DMEM without serum. After 5 min at room temperature, the two tubes were mixed and placed at room temperature for another 20 min. The mixture was added to the 12-well plate and transfected for 5 h, and then cultured for 24 and 48 h with 500 μl fresh DMEM culture medium containing 10% FBS, and received, respectively.

Data were collected as previously described in Liu et al. (2021), Dual-luciferase assays were carried out in a 96-well plate (Promega, Madison, WI, USA). The activity of renilla luciferase was normalized to that of the firefly luciferase and displayed as a percentage of the control. For each treatment, three biological replications were used.

### Statistical analysis

Expression of ssa-miR-301a-3p and *hsp90b2* was calculated in relative quantitation (RQ = 2^−ΔΔCT^). All data were analyzed using SPSS 19.0 software (IBM, Armonk, NY, USA). The relative luciferase activity value was calculated according to the formula of Renilla luciferase activity value/firefly luciferase activity value. Data of the treatment group and control group were compared using t-test, and values are expressed as mean ± standard deviation. *P*-values less than 0.05 were considered to be statistically significant and all data were shown as mean ± standard error (SE).

## Results

### Potential target gene prediction of ssa-miR-301a-3p

A total of four putative ssa-miR-301a-3p target genes were obtained by biological analysis of RNA-seq data. These four genes were *hsp90b2*, *TBCC1*, *P4HAI*, and *hsp90b1*, respectively. Among them, *hsp90b2* and *hsp90b1* were involved in heat stress-related signaling pathway, while *TBCC1* and *P4HAI* were down-regulated after heat stress. The change in expression of *hsp90b2* was much more significant than that of *hsp90b1* under heat stress (Table 2). Moreover, 3′-UTR of *hsp90b2* had a complete match with the ssa-miR-301a-3p sequence and the expression level of *hsp90b2* was significantly increased in heat stress group (HL) compared with the control group (CL). Therefore, in this study, based on the negative regulation relationship between miRNA and target genes, we chose *hsp90b2* as the target gene of ssa-miR-301a-3p. The binding site of ssa-miR-301a-3p in the 3′-UTR of *hsp90b2* was in the 247–254 position (Fig. 1).

**Table 2:** The target gene sequencing results of ssa-miR-301a-3p.

Gene name	Log_2_FoldChange	Padj
*Hsp90b2*	1.5767	1.47E−07
*TBCC1*	1.8655	0.034978
*P4HA1*	3.0113	4.41E−09
*Hsp90b1*	1.2616	0.000139

**Note:**

Log2FoldChange: log2(Sample1/Sample2), sample1 represents the readcount value of 24 °C in sequencing results, sample2 represents the readcount value of 18 °C in sequencing results; Padj: corrected pvalue. The smaller the corrected pvalue, the more significant the change in gene expression at 24 °C compared with 18 °C.

### Expression of ssa-miR-301a-3p and *hsp90b2* in the liver of rainbow trout

**Figure 2:**
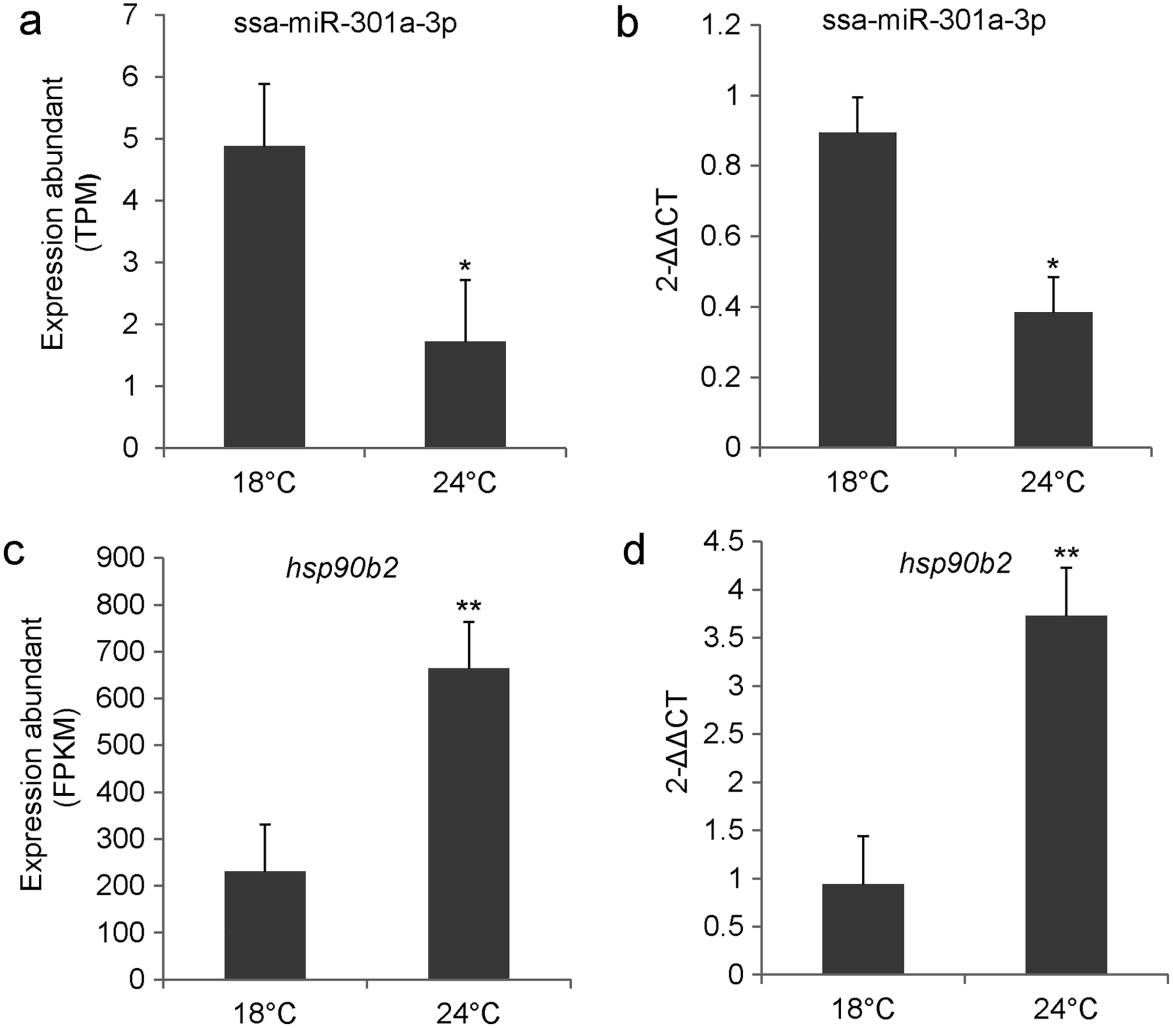
ssa-miR-301a-3p and *hsp90b2* mRNA in the liver of rainbow trout at 18 and 24 °C. Expression of ssa-miR-301a-3p and *hsp90b2* mRNA in the liver of rainbow trout at 18 and 24 °C. (A) The high-throughput sequencing result of ssa-miR-301a-3p; (B) The qRT- PCR validation result of ssa-miR-301a-3p; (C) The high-throughput sequencing result of hsp90b2; (D) The qRT-PCR validation result of *hsp90b2*. *Statistically significant difference between CL and HL *P* < 0.05. **Statistically significant difference between CL and HL *P* < 0.01.

We examined the relative expression level of ssa-miR-301a-3p and *hsp90b2* in the CL and HL by qRT-PCR. Results indicated that ssa-miR-301a-3p expression level had a significant downgrade in HL (*P* < 0.05) compared with the CL (Fig. 2B), and the relative expression of *hsp90b2* mRNA in HL was significantly up-regulated compared to the CL (*P* < 0.01) (Fig. 2D). In addition, we listed the high-throughput sequencing results of ssa-miR-301a-3p and *hsp90b2* and found that the sequencing results of qRT-PCR were basically consistent with those of high-throughput sequencing (Figs. 2A, 2C). The inverse relationship between the expressions of ssa-miR-301a-3p and *hsp90b2* mRNA in CL and HL, suggests that there might be a negative correlation between ssa-miR-301a-3p and *hsp90b2*.

### Analysis of the ssa-miR-301a-3p binding site in the 3′-UTR of *hsp90b2*

**Figure 3:**
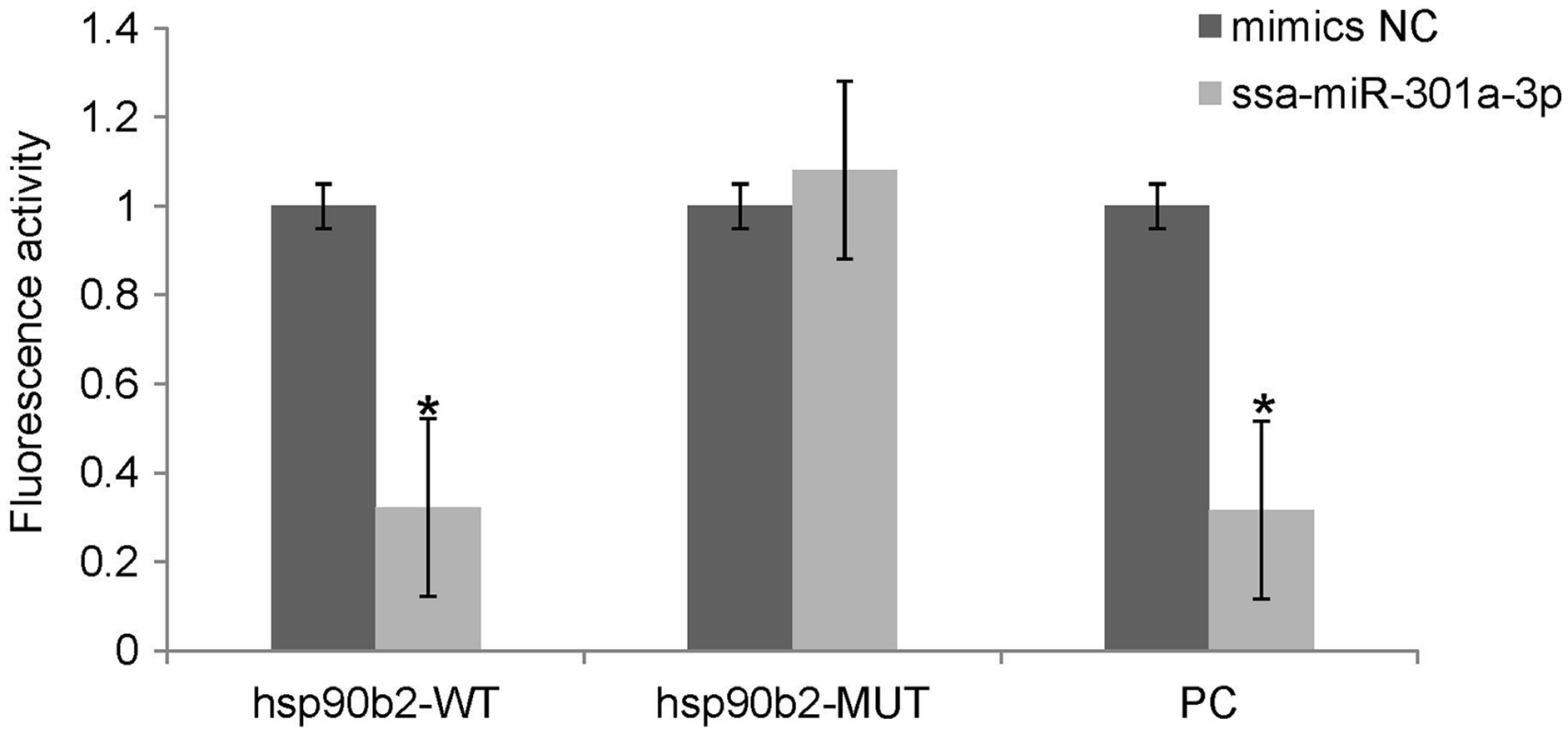
Dual-luciferase reporter assay analysis of the target relationship of ssa-miR-301a-3p with *hsp90b2* in HEK 293T cells. Relative luciferase reporter expression was normalized to NC. Each treatment was repeated in triplicates. Data are depicted as mean ± standard error. An asterisk (*) indicates *P* < 0.05 *vs* the mimic NC.

We performed luciferase activity assays using HEK 293T cells to verify whether ssa-miR-301a-3p binds to the 3′-UTR of *hsp90b2*. There was no significant change in the expression of luciferase in the mimic NC + ssa-miR-301a-3p PC (*P* > 0.05), while the co-transfection of ssa-miR-301a-3p mimic with ssa-miR-301a-3p PC revealed that luciferase activity was inhibited (*P* < 0.05) (Fig. 3), which excluded the influence of transfection agents, RNA extracts, and detection methods in the experiment. The recombinant plasmids of *hsp90b2* WT and *hsp90b2* MUT were also co-transfected into HEK 293T cells along with either the ssa-miR-301a-3p mimic or mimic NC. There was no significant change in the expression of luciferase in the mimic NC + *hsp90b2* WT and mimic NC + *hsp90b2* MUT groups (*P* > 0.05) (Fig. 3). The inhibition of luciferase activity could be found through co-transfection of ssa-miR-301a-3p mimic with *hsp90b2* WT (*P* < 0.05), but luciferase activity was not significantly affected by co-transfection of ssa-miR-301a-3p mimic and *hsp90b2* MUT (*P* > 0.05). These results demonstrated that ssa-miR-301a-3p was involved in regulation of *hsp90b2* expression by binding with the putative binding site in the 3′-UTR of the *hsp90b2*.

## Discussion

In this study, we used three methods including TargetScan, PicTa, and miRanda to predict target genes for ssa-miR-301a-3p, and found that the 3′-UTR of *hsp90b2* and complementary region of ssa-miR-301a-3p was completely complementary. We observed a significant down-regulation in luciferase activity of ssa-miR-301a-3p mimics group transfection of pmirGLO-wt-*hsp90b2* 3′-UTR; consequently, the expression of *hsp90b2* mRNA was not effectively inhibited. These data indicate ssa-miR-301a-3p can complement with the 3′-UTR site of *hsp90b2*, which makes the luciferase activity decrease significantly and ssa-miR-301a-3p participates in the heat stress response of rainbow trout by targeting *hsp90b2*. In addition, as a positive control group, ssa-miR-301a-3p mimics can significantly reduce the luciferase activity of pmirGLO-ssa-miR-301a-3p in the same experimental system, which proves that the experimental system is reasonable and the experimental results are stable and accurate. Therefore, the results showed that *hsp90b2* gene was a target gene for ssa-miR-301a-3p.

As a potential biomarker of fish’s response to thermal attack, *hsp90* provides protection for cellular internal tissues and regulates important cellular functions (Sharma, Singh & Chakrabarti, 2017). As the main cell defense against acute heat stress, HSPs can be synthesized quickly (Kregel, 2002). In this study, significantly increased expression level of *hsp90b2* was found in the liver of rainbow trout after heat stress. Like our results many other investigators also reported upregulation of *hsp90* in liver of other fish species subjected to heat shock *e.g.*, carp (Hermesz, Abraham & Nemcsok, 2001), *C. idela* (Wu et al., 2012; Sharma, Singh & Chakrabarti, 2017), *Channa striatus* (Purohit et al., 2014), *Puntius sophore* (Mahanty et al., 2017) and *Labeo Rohita, Catla Catla* (Ahmad et al., 2020). In fish, *hsp90* is involved in embryonic muscle development, metamorphosis, oxidative stress and anti-bacterial infection (Du et al., 2008; Manchado et al., 2008; Padmini & Rani, 2011; Cha et al., 2013). By participating in the aggregation of heat shock transcription factors to activate target genes, *hsp90* protects cells in heat shock conditions and also protects cells by inhibiting heat shock-induced apoptosis (Ali, Bharadwaj & Ovsenek, 1998; Yavelsky et al., 2004). Research of Rendell et al. (2006) showed that the *hsp90* synthesis in rainbow trout at different growth stages was significantly increased in the liver under heat stress. In addition, some studies have found that *hsp90b2* was involved in the endoplasmic reticulum-associated degradation (ERAD) of protein processing in endoplasmic reticulum pathway (Hirsch et al., 2009; Xie & Ng, 2010). In our previous study, GO and KEGG pathway enrichment analyses indicated that the target gene *hsp90b2* is regulated by miR-301a-3p, a process that involves the heat stress-related ER protein processing pathway (Li et al., 2017). The ubiquitin ligase Hrd1 recognizes *hsp90b2* (Bordallo et al., 1998; Bays et al., 2001), and translocation is mediated through the Cdc48-Ufd1-Npl4-p97AT pathway. Ubc7p and Hrd1p mediate ubiquitination. Finally, Rad23p and Dsk2p regulate the proteasome.

At optimum temperature (18 °C), miR-301a-3p inhibited the expression of *hsp90b2* by binding to the 3′-UTR of *hsp90b2*; while under heat stress (24 °C), miR-301a-3p induced the expression of the target gene *hsp90b2*. Furthermore, under heat stress, the number of aberrant and misfolded proteins in rainbow trout liver increases significantly. The buildup of these misfolded proteins accumulates significantly, affecting the normal function of the ER, inducing ER stress, and finally leading to cell death (Huang et al., 2018). The elimination of misfolded peptides/proteins is so critical. Under heat stress conditions, we detected a considerable downregulation of miR-301a-3p expression; as a result, *hsp90b2* expression was not successfully suppressed. These findings suggest that miR-301a-3p plays a role in rainbow trout heat stress response by targeting *hsp90b2*, which is enriched in the ERAD pathway, to increase the degradation of misfolded proteins, hence regulating the adaptive protection mechanism.

Fish growth is influenced by various aspects such as temperature variation, hypoxia, salinity and heavy metal (Myers, 1998; Topal et al., 2017). There is abundant evidence that miRNA can regulate gene regulation through direct or indirect means in the face of various kinds of stress (Bartel, 2009). Studies have shown that miRNAs have the ability to regulate the expression levels of *Hsp60* and *Hsp70* in skeletal muscle and cardiac muscle cells (Shan et al., 2010; Kukreti et al., 2013). In the present study, we found a targeted relationship between ssa-miR-301a-3p and *hsp90b2*, and when the expression of ssa-miR-301a-3p was down-regulated, the expression of *hsp90b2* was significantly up-regulated, indicating there is a negative regulatory relationship between ssa-miR-301a-3p and *hsp90b2*. When the expression of *hsp90b2* is up-regulated, the misfolding of protein may be corrected, and the immune and endocrine systems may mediate under stress conditions and resistance of rainbow trout to heat stress is enhanced.

## Conclusion

The expression level of ssa-miR-301a-3p was down-regulated and *hsp90b2* was exactly the opposite of ssa-miR-301a-3p under heat stress. There might be negative regulatory relations between ssa-miR-301a-3p and *hsp90b2* through binding with the 3′-UTR region of *hsp90b2*. Our findings provide evidence for the role of ssa-miR-301a-3p in the liver and will help understand the mechanism of miRNA-mediated gene regulation in response to heat stress in rainbow trout.

## Supplemental Information

10.7717/peerj.13476/supp-1 Supplemental Information 1 Raw data.

## Additional Information and Declarations

### Data Availability

The following information was supplied regarding data availability: The data is available at NCBI SRA: SRP109730.
