# Supplementary figures and images for: Gene ssa-miR-301a-3p improves rainbow trout (Oncorhynchus mykiss) resistance to heat stress by targeting hsp90b2
Source: PeerJ. 2022 Jul 5;10:e13476. doi: 10.7717/peerj.13476 (PMC9266697; doi:10.7717/peerj.13476)

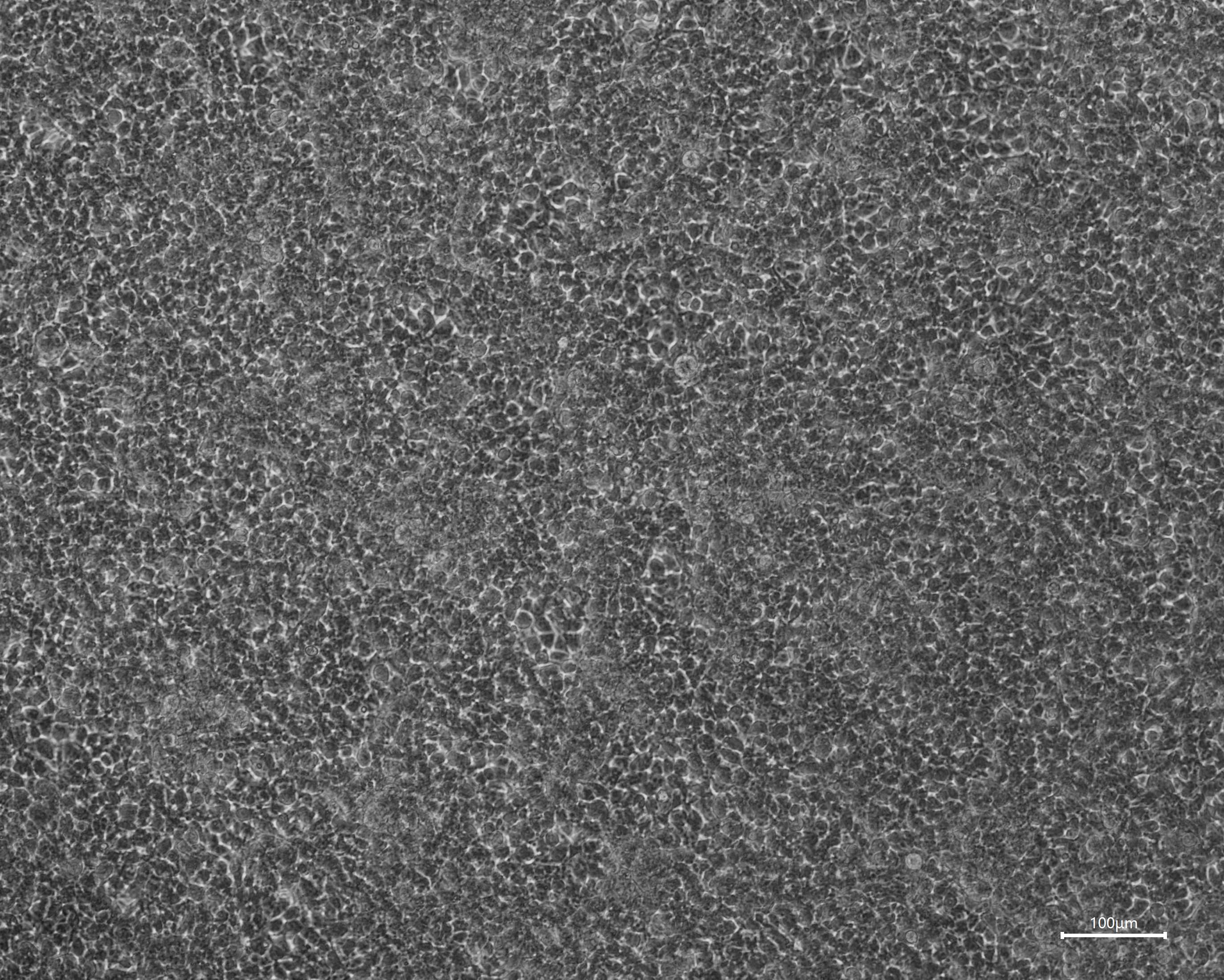

Supplement: Supplemental Information 1 [file peerj-10-13476-s001.zip › Supplemental Files/48H Received the picture/B.jpg]

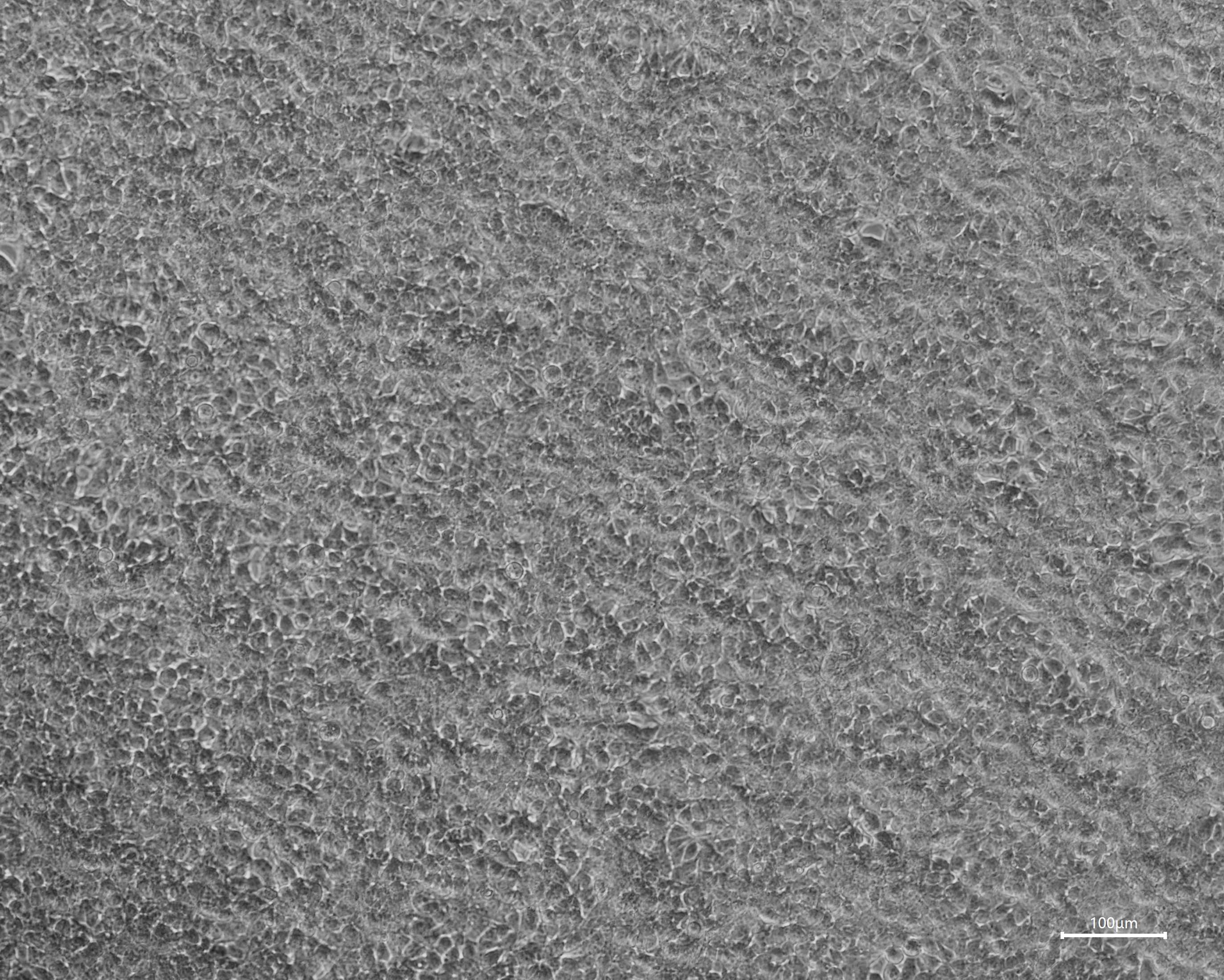

Supplement: Supplemental Information 1 [file peerj-10-13476-s001.zip › Supplemental Files/48H Received the picture/MUT+NC.jpg]

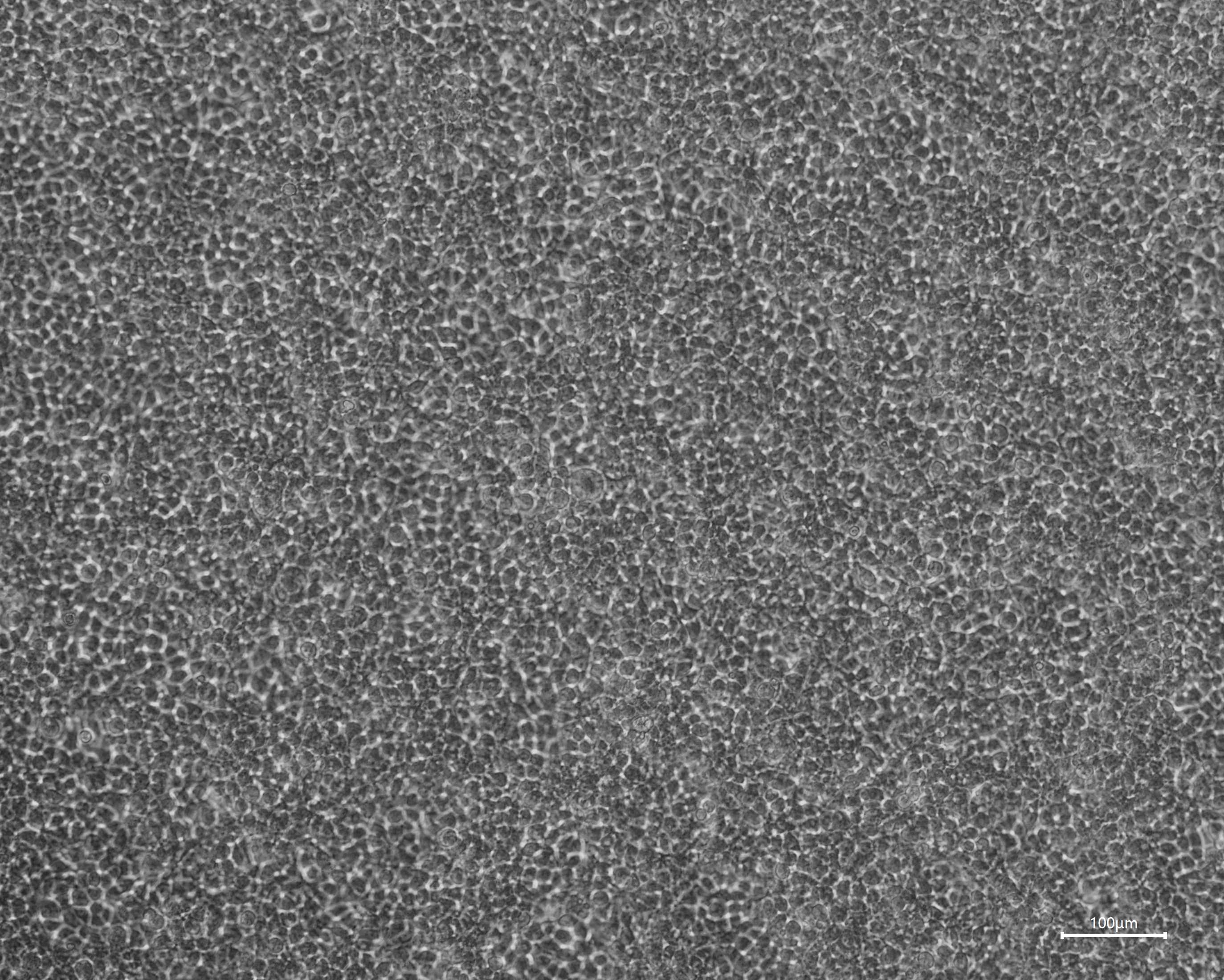

Supplement: Supplemental Information 1 [file peerj-10-13476-s001.zip › Supplemental Files/48H Received the picture/MUT+mimics.jpg]

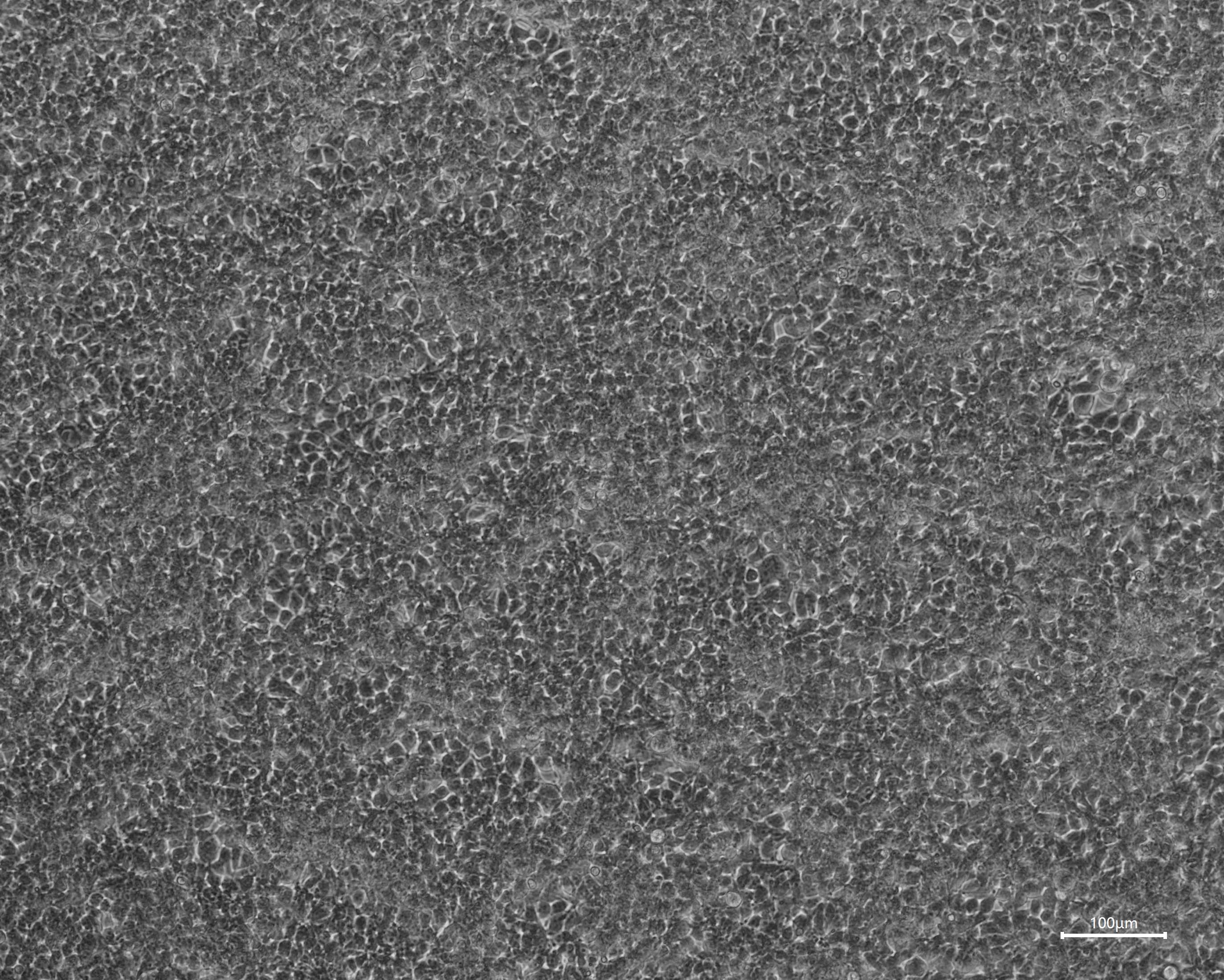

Supplement: Supplemental Information 1 [file peerj-10-13476-s001.zip › Supplemental Files/48H Received the picture/PC+NC.jpg]

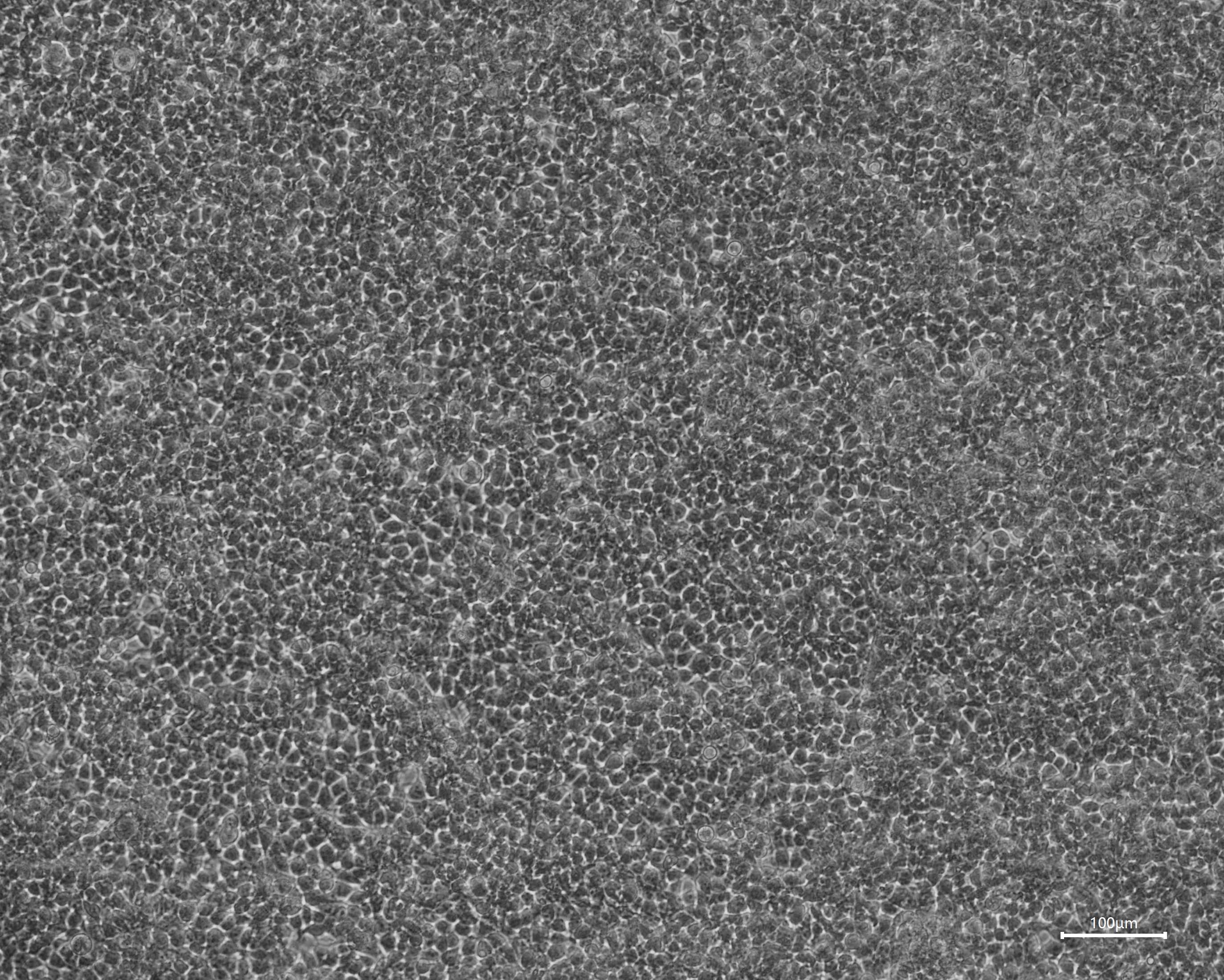

Supplement: Supplemental Information 1 [file peerj-10-13476-s001.zip › Supplemental Files/48H Received the picture/PC+mimics.jpg]

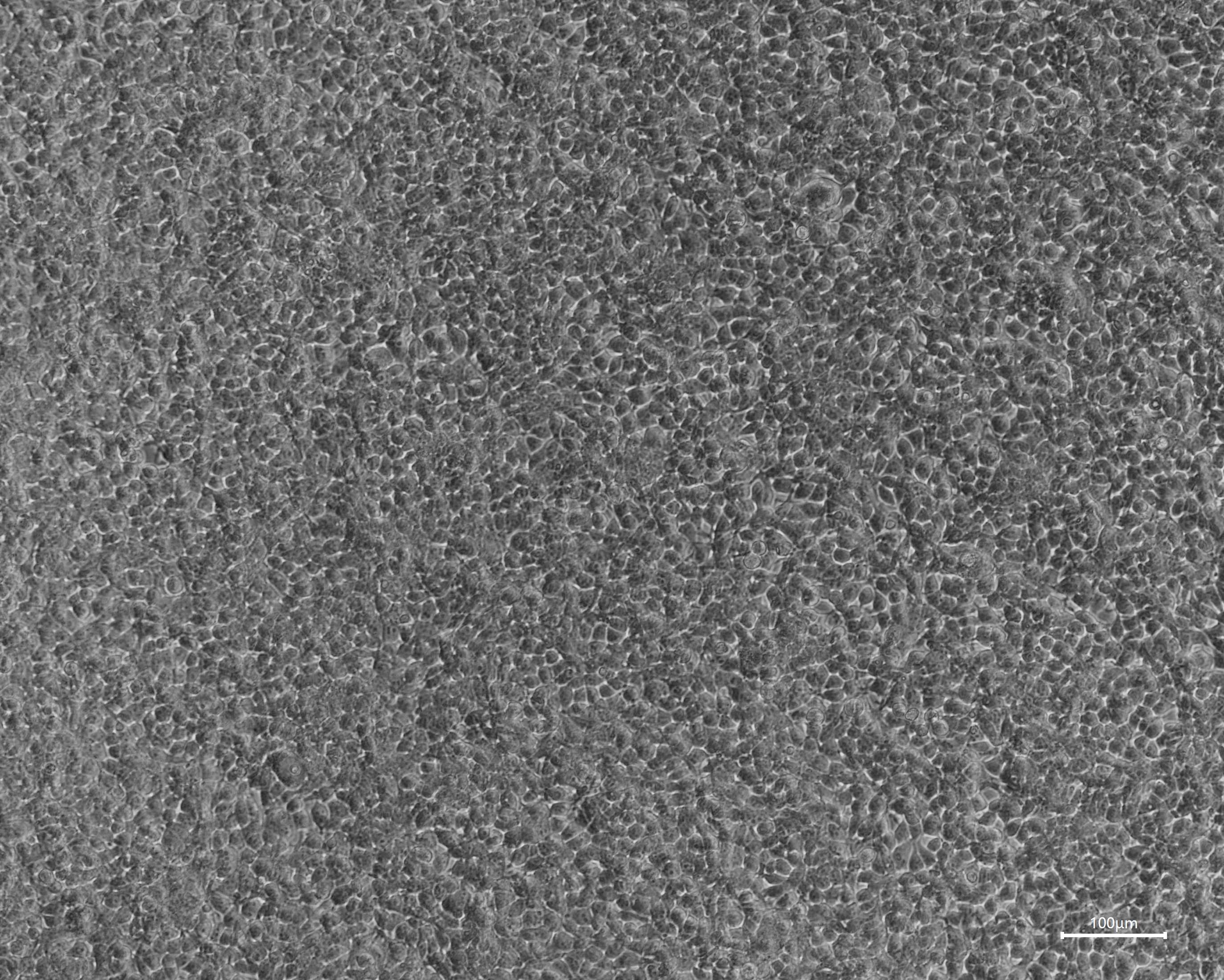

Supplement: Supplemental Information 1 [file peerj-10-13476-s001.zip › Supplemental Files/48H Received the picture/WT+NC.jpg]

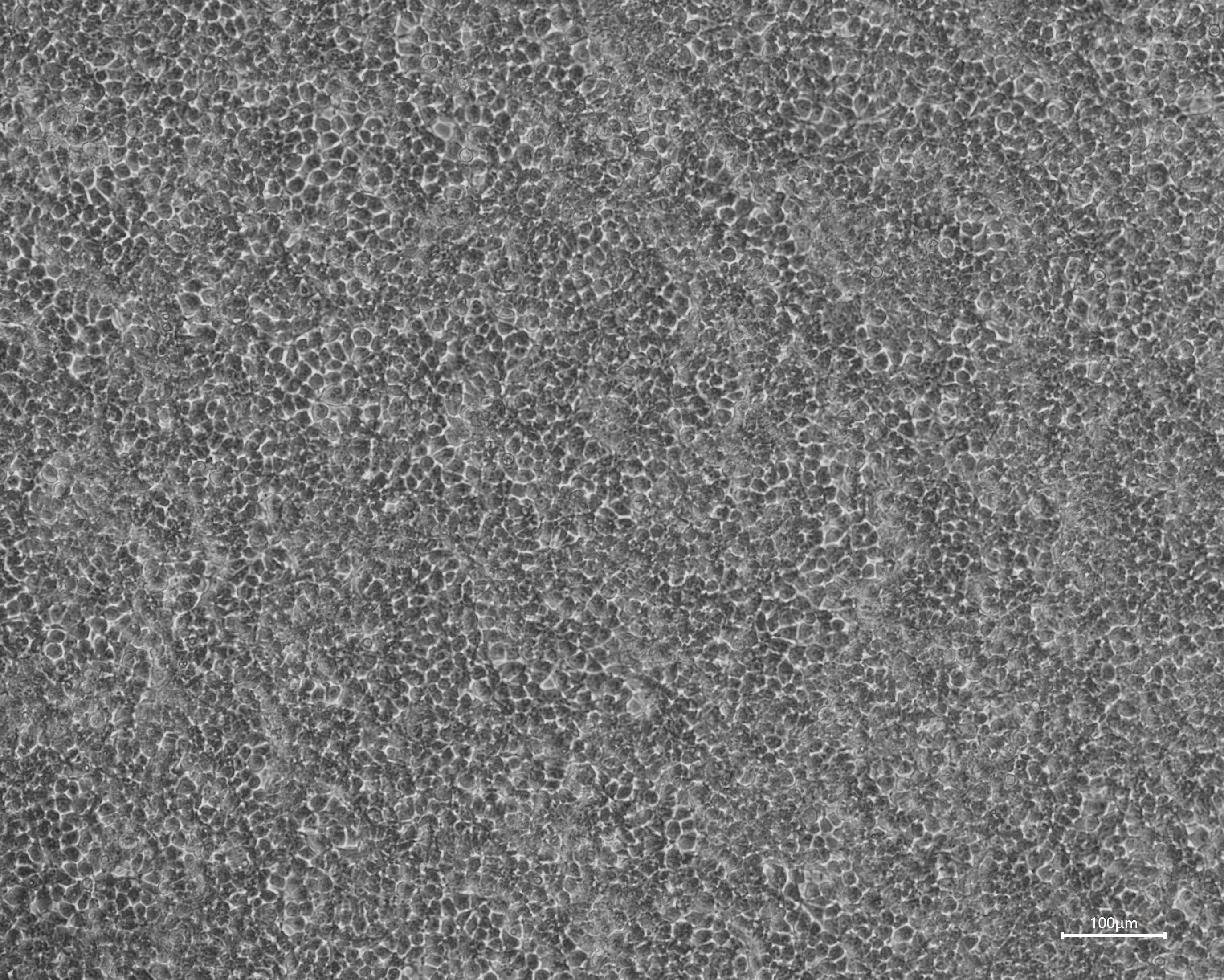

Supplement: Supplemental Information 1 [file peerj-10-13476-s001.zip › Supplemental Files/48H Received the picture/WT+mimics.jpg]
